# Adipokines as Predictive Biomarkers for Training Adaptation in Subjects with Multimorbidity—A Hypothesis-Generating Study

**DOI:** 10.3390/jcm12134376

**Published:** 2023-06-29

**Authors:** Felipe Mattioni Maturana, Rebecca Rolf, Simone Schweda, Max Reimer, Manuel Widmann, Christof Burgstahler, Andreas M. Nieß, Inga Krauss, Barbara Munz

**Affiliations:** 1 Department of Sports Medicine, University Hospital Tübingen, Hoppe-Seyler-Str. 6, D-72076 Tübingen, Germany; felipe.mattioni@med.uni-tuebingen.de (F.M.M.); rebecca.rolf@student.uni-tuebingen.de (R.R.); simone.schweda@med.uni-tuebingen.de (S.S.); max.reimer@student.uni-tuebingen.de (M.R.); manuel.widmann@med.uni-tuebingen.de (M.W.); christof.burgstahler@med.uni-tuebingen.de (C.B.); andreas.niess@med.uni-tuebingen.de (A.M.N.); inga.krauss@med.uni-tuebingen.de (I.K.); 2 Interfaculty Research Institute for Sport and Physical Activity, Eberhard Karls University of Tübingen, D-72076 Tübingen, Germany

**Keywords:** multimorbidity, obesity, physical exercise, adipokines, leptin

## Abstract

Background. Physical exercise exerts a positive effect on many chronic conditions, specifically lifestyle-related diseases such as overweight and obesity, type 2 diabetes mellitus (T2DM), cardiovascular conditions and osteoarthritis (OA). As a result of common risk factors, most of these patients present with multiple conditions. Exercise- and disease-related biomarkers, such as adipokines, are emerging tools in training supervision and regulation; however, their significance in subjects with multimorbidities is unknown. Subjects and Methods. To address this issue, adipokines leptin, adiponectin and resistin were assessed in a cohort of subjects with multimorbidities (n = 39) presenting with at least two of the abovementioned conditions or relevant risk factors before and after a six-month exercise and lifestyle intervention program (‘MultiPill-Exercise’), and correlated with training adaptation, namely changes in relative maximum oxygen uptake (${\overset{\cdot }{V}O}_{2}\max$). Results. There was a significant negative correlation between baseline leptin concentrations and training effect for relative ${\overset{\cdot }{V}O}_{2}\max$ (after three months: rho = −0.54, *p* = 0.020 *; after six months: rho = −0.45, *p* = 0.013 *), with baseline leptin explaining 35% of the variance in delta relative ${\overset{\cdot }{V}O}_{2}\max$ after three months and 23% after six months. Conclusions. Leptin might be a suitable surrogate biomarker in the context of exercise-based lifestyle intervention programs in subjects with multimorbidity.

## 1. Introduction

The prevalence of lifestyle-related diseases, namely overweight and obesity, type 2 diabetes mellitus (T2DM), cardiovascular conditions and osteoarthritis (OA), is high and still increasing worldwide [1,2,3,4]. As a result of overlapping risk factor profiles, most subjects are eventually diagnosed with more than one of these conditions or display relevant risk factors for a second (or third, fourth, etc.) disease. Thus, multimorbidity is the rule, not just the exception, in this context.

Multimorbidity is a complex phenomenon, for which, to date, no generally accepted definition exists. While the WHO defines multimorbidity as ‘the co-occurrence of two or more chronic medical conditions in one person’, this definition is not very useful in clinical practice, where usually a narrower definition, specifically focusing on lifestyle-related diseases, is more appropriate. While typical disease combinations and patterns can be defined, for example, T2DM in combination with obesity, it is important to note that a ‘typical’ multimorbid patient does not exist, which has to be taken into account when developing and designing therapeutic strategies. In addition, associated factors, namely psychosocial characteristics (see below) or medication—multimorbidity is often accompanied with polypharmacy—have to be taken into account (for review, see [5]).

The high prevalence of lifestyle-related diseases, usually in the context of multimorbidity, has led to the development of a new field of medicine, the so-called ‘lifestyle medicine’. It analyzes the complex interplay of different characteristics of an unhealthy lifestyle and their contribution to disease development. In addition, based on this knowledge, it develops strategies aiming at attenuating lifestyle-associated risk factors. These can act on a preventive or therapeutic level. The most important risk factors associated with an unhealthy lifestyle are tobacco smoking, overweight/obesity, a high blood pressure, an unhealthy diet and a sedentary lifestyle. Most patients with chronic lifestyle-related diseases present with several of these risk factors; thus, programs aiming at helping these patients to adopt a healthier lifestyle should be multi-faceted, including elements to enhance physical activity as well as nutritional counselling. In addition, psychosocial factors, such as volition and motivation, are very important. Consequently, lifestyle intervention programs should also contain coaching sessions that help patients overcome typical barriers and obstacles that might hamper the adoption of a healthier lifestyle and—even more importantly—their adherence to it (for review, see [6]).

Nevertheless, despite the fact that lifestyle intervention programs based on these elements, i.e., the promotion of physical activity as well as nutritional and psychosocial counselling, are known to be highly efficient to prevent, treat and stabilize lifestyle-related diseases (for review, see [6]), so far, exercise responses in subjects with multimorbidity have not been well characterized, and it is unclear how training programs for this cohort of patients should be designed, monitored and regulated [7].

Exercise biomarkers, i.e., biological factors that respond to physical activity and sports and can be assessed in bodily fluids such as blood or urine, are promising candidates for training supervision and management. In the context of subjects with lifestyle-related diseases, metabolism-associated markers in particular have important potential. Adipokines such as leptin, adiponectin and resistin are released from adipose tissue and are involved in metabolic control. All of these markers have been shown to respond to exercise interventions in patients with either (pre)diabetes and/or overweight/obesity (for reviews, see [8,9]).

‘MultiPill-Exercise’ is a lifestyle intervention program designed to increase exercise participation and improve activity behavior in subjects with multimorbidity. The program consists of a set of exercise-based modules, as well as associated elements such as psychosocial counselling or nutritional support, and has previously been described in detail [10].

Briefly, the program consisted of two 12 week periods, the first of which was designed as a more supervised phase, including regular sessions at our outpatient clinic. Here, patients underwent a specific training program, consisting of different elements, where all patients were subjected to individually shaped sessions of endurance, strength and functional training, in which training intensity was continuously adapted with increasing fitness. The program also included so-called ‘movement teasers’, in which patients could choose between a variety of different activities, such as Thai Chi/Yoga, Aqua Fitness or dancing. This first 12 week period was followed by a more self-directed period of the same duration, during which subjects were advised to maintain regular physical activity while training at local gyms or recreational fitness centers. In addition, patients were also advised to keep a training log. The program also contained classroom sessions, delivering both theoretical and practical knowledge on how to achieve a healthy lifestyle. These lectures addressed topics such as ‘principles of exercise and training’, ‘active everyday lifestyle’ and ‘nutrition and health’, as well as psychosocial factors such as personal motives and goals, motivation, volition and barriers. Furthermore, patients were offered individual counselling sessions to address the topics most relevant to them in more depth, as well as additional disease-specific offerings such as relaxation classes, workshops teaching strengthening exercises for specific muscles or T2DM-oriented nutritional counselling.

The data described here are the results of a pilot study on n = 39 subjects that was carried out in 2019/2020 [11]. Based thereon, a prospective, randomized, controlled trial on n = 320 subjects was initiated in April 2022 [11].

To assess the potential relevance of exercise-responsive biomarkers in this cohort, we tested three adipokines (leptin, adiponectin and resistin). The primary goals of our exploratory study were to assess adipokine patterns in this heterogeneous patient subgroup throughout the intervention to evaluate their potential to monitor and predict individual training responses, and to establish specific hypotheses for further testing in the future.

## 2. Subjects, Materials and Methods

Subjects and training intervention. The design of our pilot study ‘MultiPill-Exercise’ has previously been described [12]. Briefly, n = 39 subjects (men: n = 12; women: n = 27; mean age 55.2 ± 10.3 years; mean BMI 31.1 ± 3.0 kg/m^2^) who presented with at least two of four conditions (obesity, T2DM, cardiovascular disease and OA or a risk thereof) were enrolled in the study. Subjects were recruited in two waves, starting in August of 2019 (n = 20) and in January of 2020 (n = 19). They were exposed to a six-month health intervention program which included endurance and resistance physical activity (PA), as well as psychosocial and nutritional counselling. At baseline (T0), as well as after three (T1) and six (T2) months of the intervention, extensive diagnostics, including spiroergometry and calipometry (three-point method [13,14]), as well as blood and urine sampling, were performed. Urine was taken to assess a specific OA marker, i.e., CTX-II (C-terminal cross-linked telopeptide of type II collagen); however, due to the low number of OA patients in our sample, a statistical analysis of these data was not possible, similar to data for another OA marker, COMP (cartilage oligomeric protein), that we assessed in the circulation. Due to COVID-19-associated restrictions, T1 diagnostics had to be cancelled for subjects in wave #2. In addition, as specified in [11], the intervention itself had to be considerably adapted and modified for this group to comply with COVID-19 contact restrictions then effective at our institution. For some subjects, no spiroergometry data were available at T1 or T2, or they dropped out of the entire study (for reasons, see [11]). In addition, for technical reasons, some or all biomarkers were not determined for certain subjects included in wave #2. For details, see Table 1. The study was conducted in accordance with the Declaration of Helsinki, approved by the Ethics Committee of the Medical Clinic Tübingen (298/2019BO2, 05-04-2019) and registered at the German clinical trial register (DRKS00016702). For a flow diagram of study progression, please refer to [11].

Determination of adipokine concentrations. Adipokines (leptin, adiponectin and resistin) were determined using specific ELISAs (leptin: E07 ELISA/Mediagnost, Reutlingen, Germany; adiponectin: E09 ELISA/Mediagnost, Reutlingen, Germany; resistin: E 50 ELISA/Mediagnost, Reutlingen, Germany). All factors were determined from patients’ serum. At baseline (T0), n = 39 samples of waves #1 and #2 were analyzed for adiponectin and resistin and n = 38 for leptin (no data available for MP1952 for technical reasons). After three months (T1) and after six months (T6), n = 20 and n = 17 samples, respectively, of the first wave were analyzed for all markers, with n = 3 subjects (MP1902, MP1912 and MP1913) having been lost to follow-up at T2. In addition, for technical reasons, no biomarkers were assessed for subjects of wave #2 at T2. For leptin analyses, sera of MP1932, MP1946 and MP1949 had to be diluted two-fold, in contrast to all other samples which were employed in the assays as undiluted fluids. With the exception of the leptin reading for subject MP1953, which corresponded to a single read, all analyses were run in duplicates and the respective means were taken for further analysis. Finally, for subjects MP1906 and MP1913, no spiroergometry data were available at T1, and for subjects MP1901 and MP1919 at T2, since subjects discontinued cycling for reasons other than peripheral exhaustion. For details, see Table 1.

Statistical analysis. Quantitative results were reported as means ± standard deviation. Comparisons of biomarker concentrations between patient subgroups and within-group comparisons (i.e., over time) were carried out using unpaired or paired *t*-tests, respectively. Simple linear regression and multiple linear regression analyses were performed to analyze the data through generalized least squares. Additionally, Akaike information criterion (AIC) was used to compare models through the maximum likelihood estimate of each model, with lower levels indicating better fits of the respective model. Shapiro–Wilk tests were performed on each variable to test its normality, and in case it was significant (*p* < 0.05), a log transformation was applied to the data. Spearman’s correlation coefficients were used to determine the association between two variables. Due to the exploratory character of our study, no correction for multiple testing was introduced. Statistical analyses were carried out using SPSS, Version 26.0 (IBM Corporation, Armonk, New York, NY, USA).

## 3. Results

### 3.1. Body Weight, Body Fat and Waist Circumference

Body weight, body fat and waist circumference did not change significantly during the intervention (Supplementary Table S1).

### 3.2. Adipokine Concentrations in the Different Patient Subgroups throughout the Intervention

When baseline biomarker concentrations were assessed, we detected elevated leptin levels in subjects with obesity or a risk thereof (no obesity: 10.5 ± 5.6 ng/mL; obesity (risk): 26.8 ± 9.9 ng/mL, *p* = 0.009 **; obesity: 45.5 ± 27.8 ng/mL, *p* = 0.021 *; Supplementary Figure S1). In parallel to the intervention, leptin levels significantly declined in this group (T1: 40.3 ± 32.3 ng/mL, *p* = 0.021 *; T2: 24.1 ± 14.7 ng/mL; *p* = 0.006 **). In contrast, adiponectin and resistin levels did not change significantly during the intervention (Supplementary Figure S1).

### 3.3. Changes in Adipokine Concentrations during the Intervention

Next, we analyzed and compared adipokine concentrations in individual subjects and their changes throughout the intervention. As shown in Supplementary Figure S2, leptin levels showed a high degree of inter-individual variability at baseline, with a tendency of higher levels in subjects at risk for obesity or T2DM. Nevertheless, most subjects, specifically those with very high leptin levels, displayed decreasing leptin levels in parallel to the intervention. For certain subjects, this was obvious at T1, whereas for others, the effects could only be detected at T2. In contrast, for adiponectin and resistin concentrations, inter-individual variability did not considerably differ when baseline, T1 and T2 patterns were compared (Supplementary Figure S2).

### 3.4. Correlation of Adipokine Profiles with (Changes in) Endurance Capacity (Delta ${\overset{\cdot }{V}O}_{2}\max$)

#### 3.4.1. Baseline Adipokine Profiles in the Context of Baseline ${\overset{\cdot }{V}O}_{2}\max$ (‘Baseline-Baseline’)

**Figure 1:**
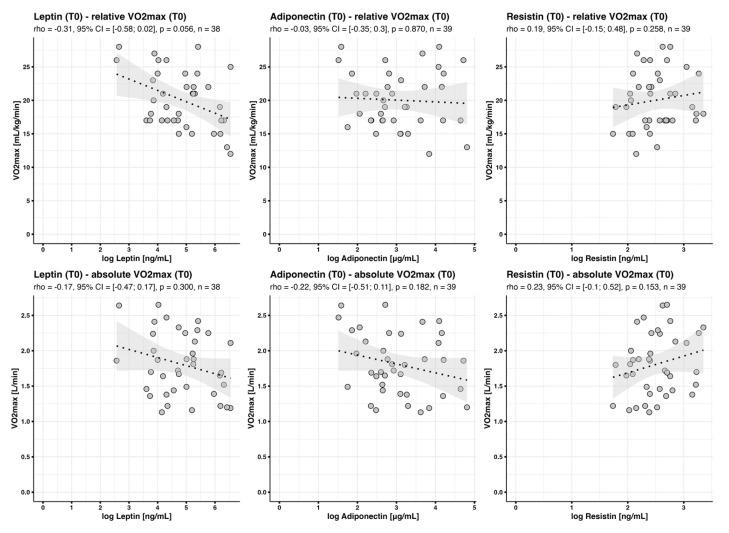
Correlation between baseline adipokine concentrations and baseline ${\overset{\cdot }{V}O}_{2}\max$. Scatter plots with fitted regression line and 95% confidence interval band show correlations between adipokine concentrations at baseline and baseline (relative and absolute) ${\overset{\cdot }{V}O}_{2}\max$.

Although, as shown in Figure 1, there was no correlation between baseline leptin concentrations and baseline relative ${\overset{\cdot }{V}O}_{2}\max$ (rho = −0.31, *p* = 0.056), baseline leptin concentrations predicted 14% of baseline variance in relative ${\overset{\cdot }{V}O}_{2}\max$. These data suggest a close relationship between leptin concentrations and ${\overset{\cdot }{V}O}_{2}\max$. However, leptin concentration was a much weaker predictor of cardiorespiratory fitness when absolute instead of relative ${\overset{\cdot }{V}O}_{2}\max$ was analyzed: Baseline leptin concentrations only predicted 8% of baseline variance in ${\overset{\cdot }{V}O}_{2}\max$-abs. Moreover, baseline adiponectin and resistin concentrations did not correlate with baseline fitness (Figure 1).

#### 3.4.2. Baseline Adipokine Profiles in the Context of Changes in ${\overset{\cdot }{V}O}_{2}\max$ (‘Baseline-Delta’)

Our primary goal was to evaluate whether baseline adipokine levels might be predictors of subsequent training-induced gains in aerobic fitness. Overall, we observed a significant increase in maximum relative oxygen consumption (${\overset{\cdot }{V}O}_{2}\max$) between T0 and T1 (2.6 mL/kg/min, *p* < 0.001) and T0 and T2 (2.0 mL/kg/min, *p* = 0.001) [11].

**Figure 2:**
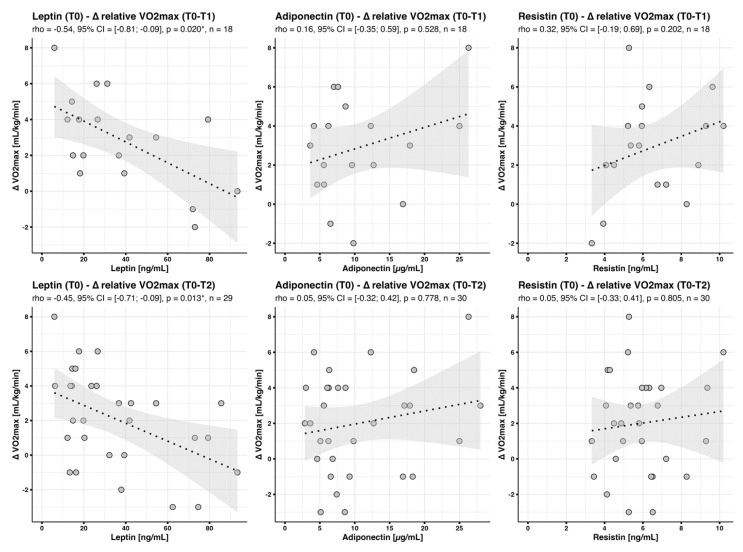
Correlation between baseline adipokine concentrations and changes in ${\overset{\cdot }{V}O}_{2}\max$. Scatter plots with fitted regression line and 95% confidence interval band show correlations between adipokine concentrations at baseline and changes in (relative) ${\overset{\cdot }{V}O}_{2}\max$. * *p* < 0.05.

There was a moderate but significant negative correlation between baseline leptin concentrations and training effect for relative ${\overset{\cdot }{V}O}_{2}\max$ (delta T0-T1: rho = −0.54, *p* = 0.020 *; delta T0-T2: rho = −0.45, *p* = 0.013 *, Figure 2). Moreover, baseline leptin explained 35% of the variance in delta relative ${\overset{\cdot }{V}O}_{2}\max$ between T0 and T1 (AIC = 82.14), and 23% of the variance between T0 and T2 (AIC = 138.85). These data suggest a close relationship between leptin concentrations and training-induced changes in relative ${\overset{\cdot }{V}O}_{2}\max$. Furthermore, as seen above for baseline ${\overset{\cdot }{V}O}_{2}\max$ values, there was little association of adiponectin and resistin baseline levels with delta ${\overset{\cdot }{V}O}_{2}\max$ (Figure 2).

#### 3.4.3. Changes in Adipokine Profiles in the Context of Changes in ${\overset{\cdot }{V}O}_{2}\max$ (‘Delta-Delta’)

**Figure 3:**
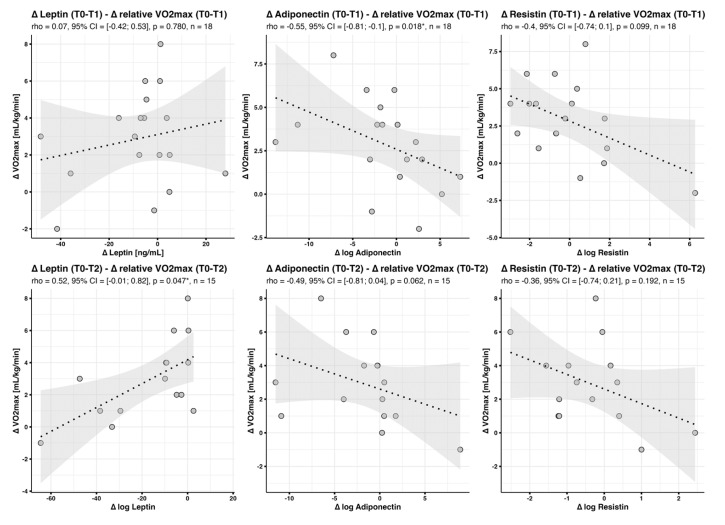
Correlation between changes in adipokine concentrations and changes in ${\overset{\cdot }{V}O}_{2}\max$. Scatter plots with fitted regression line and 95% confidence interval band show correlations between changes in adipokine concentrations and changes in (relative) ${\overset{\cdot }{V}O}_{2}\max$. * *p* < 0.05.

When we analyzed adaptations in biomarker concentrations in the context of training effects, moderate, but significant, correlations were found between changes in leptin concentrations and relative delta ${\overset{\cdot }{V}O}_{2}\max$ (rho = 0.52, *p* = 0.047 *) between T0 and T2, and changes in adiponectin and relative delta ${\overset{\cdot }{V}O}_{2}\max$ (rho = −0.55, *p* = 0.018 *) between T0 and T1 (Figure 3).

**Figure 4:**
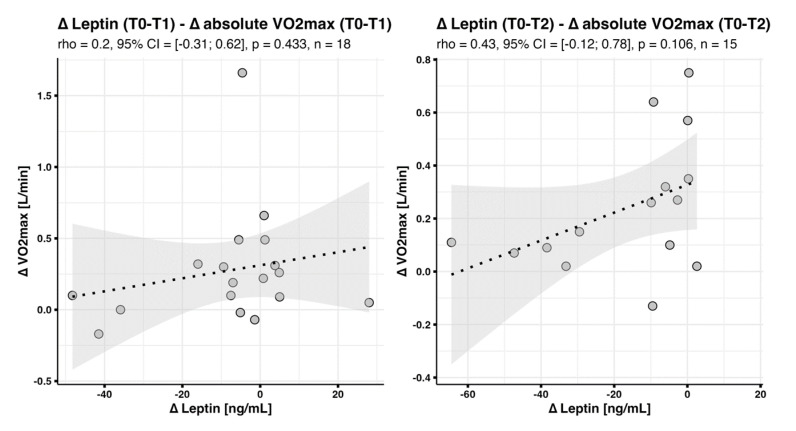
Correlation between changes in leptin concentrations and changes in absolute ${\overset{\cdot }{V}O}_{2}\max$. Scatter plots with fitted regression line and 95% confidence interval band show correlations between changes in leptin concentrations and changes in absolute ${\overset{\cdot }{V}O}_{2}\max$.

Again, these correlations could no longer be detected when changes in absolute instead of relative ${\overset{\cdot }{V}O}_{2}\max$ were studied; delta leptin (T0-T1) predicted as little as 4% of delta ${\overset{\cdot }{V}O}_{2}\max$-abs (T0-T1), and delta leptin (T0-T2) only 19% of delta ${\overset{\cdot }{V}O}_{2}\max$-abs (T0-T2) (Figure 4).

### 3.5. Leptin, BMI and Body Fat

As expected, leptin concentrations and BMI were strong confounders (leptin (T0)—BMI (T0): rho = 0.66, *p* < 0.001 ***; BMI (T0)—delta leptin (T0-T1): rho = −0.44, *p* = 0.054; BMI (T0)—delta leptin (T0-T2): rho = −0.59, *p* = 0.013 *; delta BMI (T0-T1)—delta leptin (T0-T1): rho = 0.62, *p* = 0.003 **; delta BMI (T0-T2)—delta leptin (T0-T2): rho = 0.65, *p* = 0.005) (Supplementary Figure S3) with BMI explaining 42% of the variance in leptin concentrations at T0; delta BMI (T0-T1), predicting 40% of delta leptin (T0-T1); and delta BMI (T0-T2), predicting 35% of delta leptin (T0-T2). At least for the T2 time point, this was reflected in a significant (negative) association between BMI and training-induced changes in relative (delta T0-T1: rho = −0.38, *p* = 0.122; delta T0-T2: rho = −0.41, *p* = 0.025 *) but not absolute ${\overset{\cdot }{V}O}_{2}\max$ (delta T0-T1: rho = −0.13, *p* = 0.616; delta T0-T2: rho = −0.29, *p* = 0.120) (Supplementary Figure S4). Additionally, baseline BMI explained 22% of the variance in delta relative ${\overset{\cdot }{V}O}_{2}\max$ between T0 and T1 (AIC = 85.42), and 25% between T0 and T2 (AIC = 142.63). In contrast, body fat was a poor predictor of training effect, explaining only 13% (AIC 87.4) and 8% (AIC 149.1) of variability between relative ∆${\overset{\cdot }{V}O}_{2}\max$, and 0% (AIC 23.6) and 1% (AIC 10.8) of variability between absolute delta ${\overset{\cdot }{V}O}_{2}\max$ (Supplementary Figure S5), although this might in part be attributed to the low precision of the calipometry method, as reflected by the high and inconsistent intra-individual variability over time (Supplementary Figure S6).

## 4. Discussion

Adipokine leptin was identified in 1994 as the gene product of the *ob* gene, which is defect in obese *ob/ob* mice. Via modulation of the production of specific neuropeptides, it regulates the activity of the ‘hunger’ and ‘satiety’ centers in the hypothalamus, thereby repressing appetite and consequently food intake. Thus, initially, strategies aiming at increasing leptin levels were considered as a new therapeutic approach in the treatment of obesity. However, it was soon discovered that individuals with obesity show increased, not decreased, levels when compared to lean subjects, despite the fact that leptin deficiency leads to obesity in *ob/ob* mice. The reason for this is that people with obesity have often developed resistance to the actions of leptin, a complex phenomenon involving, among others, effects on leptin transport, the density of leptin receptors on cell surfaces and leptin receptor downstream signaling. Novel therapeutic approaches aim at combining leptin administration with that of a so-called leptin sensitizer, which can overpower leptin resistance (for a review, see [15]). In addition, as demonstrated in our study, leptin might be a suitable predictive biomarker in the context of exercise-centered lifestyle intervention programs for subjects with obesity.

In the context of clinical practice, our data indicate that leptin might be a suitable surrogate biomarker to manage, monitor and evaluate exercise-based lifestyle interventions for subjects with multimorbidity, whereas adiponectin and resistin might be less appropriate. Particularly in subjects at risk for obesity or T2DM, adiponectin and resistin concentrations did not change significantly throughout the intervention. In contrast, as expected, leptin concentrations were elevated in subjects with overweight and obesity, and, specifically in the latter, declined during the intervention. A qualitative individualized analysis suggested that, predominantly in subjects with very high leptin readings at baseline, concentrations of this adipokine strongly declined by T1 and (even more) T2. Moreover, we observed a strong association between baseline leptin levels and changes in cardiorespiratory fitness (${\overset{\cdot }{V}O}_{2}\max$), indicating that people with higher leptin concentrations benefitted less from the intervention in terms of increasing their aerobic capacity. This effect might in part be due to the fact that ${\overset{\cdot }{V}O}_{2}\max$ and BMI, as well as BMI and leptin concentrations, are strong confounders, as has been described in a broad variety of other previous studies (for reviews, see [16,17,18]). Consistently, in our study population, there was a positive correlation between changes in leptin levels and changes in BMI during the intervention, illustrating that weight loss was immediately associated with decreasing leptin levels. In contrast, there was no negative correlation between intervention-induced changes in ${\overset{\cdot }{V}O}_{2}\max$ and changes in leptin levels, indicating that the respective effects might be more long term. In general, the observed associations between BMI and training effects might be explained by the facts that (1) our intervention was not controlled for exercise duration and intensity, so that it is well possible that people with a lower BMI might have trained more efficiently and (2) we assessed relative ${\overset{\cdot }{V}O}_{2}\max$, so that gains in absolute ${\overset{\cdot }{V}O}_{2}\max$ might have been underestimated in subjects with a high BMI. The latter hypothesis is supported by the finding that the negative correlations between baseline BMI or leptin levels and changes in absolute ${\overset{\cdot }{V}O}_{2}\max$ were much weaker when compared to correlations with changes in relative ${\overset{\cdot }{V}O}_{2}\max$, and mostly non-significant.

With regard to training practice, the fact that baseline leptin levels, explaining 35% (AIC 82.54)/22% (AIC 138.85) of changes in relative ${\overset{\cdot }{V}O}_{2}\max$ between T0 and T1 or T0 and T2, were a better predictor of training effects than baseline BMI, explaining 22% (AIC 85.42)/25% (AIC 142.63), respectively, is noteworthy, suggesting that leptin concentrations might be superior to BMI in predicting individual training success, probably due to the fact that they more closely reflect body fat. Not only are adipokines mainly produced in adipose tissue (for review, see [19]), but they also reflect its distribution. Specifically, subcutaneous adipocytes secrete higher levels of leptin than those of the visceral type (for review, see [20]). It is possible that, for issues related to mobility, for people with more subcutaneous fat, exercising efficiently might be harder when compared to individuals with a higher proportion of visceral fat, thus leading to lower gains in fitness. In addition, there might be gender- and age-related effects, since fat distribution is different in men and women and also dependent on age [20], which might be correlated with different training efficiencies in different patient subgroups. In addition, several studies suggest that, independently of fat distribution, there might be gender-specific differences in regard to leptin regulation (for a review, see [15]). Unfortunately, due to the low number of male subjects in our sample, it was not possible to assess gender-specific differences with regard to correlations between leptin concentrations and training response with sufficient statistical reliability. However, interestingly, a preliminary analysis suggests that a correlation between baseline leptin and training response might be particularly strong in women (Supplementary Figure S7). This will be analyzed in subsequent studies in more detail.

Moreover, all known methods to assess body fat are either inexact and time consuming, such as skinfold calipometry used in our study, or require very expensive and complex equipment (for a review, see [21]), warranting the need for novel strategies. It is very likely that leptin might be an even better predictor of training adaptation in subject cohorts with a higher degree of variability with regard to body composition. Consequently, in the future, it might be very promising to study the potential of leptin concentration as a biomarker for individualized training control in more detail, particularly in different patient subgroups. Furthermore, when analyzing larger cohorts, it will also be possible to include patients with higher degrees of multimorbidity, including a broader spectrum of chronic diseases, namely psychiatric conditions such as depression or cancer.

Thus, in the future and specifically in subjects with complex diagnoses and high degrees of multimorbidity, assessing leptin levels prior to starting medical training interventions might be a simple, quick and cost-effective way to predict individual responses to sports programs. Based on this assumption, further studies aimed at designing effective training programs for such potential ‘low responders’ might be carried out. It is likely that for a training regimen to be effective in such subjects, it will have to be more restrictive and closely controlled when compared to regimens for patients predisposed to respond well to exercise.

Taken together, our data suggest that—in addition to BMI—leptin concentrations might be a predictive marker for the effects of an exercise-based lifestyle intervention program on the cardiorespiratory fitness in subjects with multimorbidity. Against this background, it might be an effective strategy to enroll high-BMI and/or high-leptin subjects particularly in highly supervised, intensity-controlled exercise regimens.

Limitations of our study: Our study was a pilot, hypothesis-generating study with only n = 39 subjects. In addition, due to COVID-19-associated contact restrictions, there were no T1 data for subjects recruited in wave #1, thus further decreasing numbers. In addition, our study did not contain a control group. Consequently, data will have to be verified in larger and more heterogeneous cohorts. To this end, a larger clinical trial, the MultiPill-Exercise main study, a randomized controlled trial, was initiated in April 2022.

## Figures and Tables

**Table 1:** Summary of data availability throughout the study. Data availability is listed for individual subjects at three different time points. The green color indicates available samples, and red, blue and yellow indicate missing data as indicated. BM: biomarkers.

			Spiroergometry, Clinical Parameters, Blood/Urine		
			T0	T1	T2
**wave 1**	1	MP1901			no spiroergometry
	2	MP1902			no data
	3	MP1903			
	4	MP1905			
	5	MP1906		no spiroergometry	
	6	MP1907			
	7	MP1910			
	8	MP1911			
	9	MP1912			no data
	10	MP1913		no spiroergometry	no data
	11	MP1914			
	12	MP1915			
	13	MP1916			
	14	MP1917			
	15	MP1918			
	16	MP1919			no spiroergometry
	17	MP1922			
	18	MP1923			
	19	MP1924			
	20	MP1925			
**wave 2**	21	MP1927			BM not analyzed
	22	MP1928			no data
	23	MP1930			BM not analyzed
	24	MP1932			BM not analyzed
	25	MP1933			no data
	26	MP1934			BM not analyzed
	27	MP1937			BM not analyzed
	28	MP1939		no T1 diagnostics	no data
	29	MP1940		due to	BM not analyzed
	30	MP1942		COVID19	BM not analyzed
	31	MP1944		lockdown	no data
	32	MP1946			BM not analyzed
	33	MP1947			BM not analyzed
	34	MP1948			BM not analyzed
	35	MP1949			BM not analyzed
	36	MP1950			BM not analyzed
	37	MP1951			BM not analyzed
	38	MP1952	no leptin		BM not analyzed
	39	MP1953			BM not analyzed

## Data Availability

All data are available on request from the corresponding author.

## Supplementary Materials

The following supporting information can be downloaded at: https://www.mdpi.com/article/10.3390/jcm12134376/s1: Figure S1. Adipokine concentrations at baseline (T0), T1 and T2 in the different patient subgroups. Columns represent means ± SD. Statistically significant differences are indicated (* *p* < 0.05, ** *p* < 0.01). Figure S2. Individual time course of adipokine concentrations for patients of the first wave (n = 20). Diagrams illustrate changes in adipokine concentrations in individual subjects over time. Dark yellow lines represent subjects with obesity (BMI > 30), light yellow lines represent “at risk” subjects (BMI 27–30) and grey lines represent non-affected subjects (BMI < 27). Similarly, dark green lines represent subjects with T2DM, light green represent “at risk” and grey lines represent non-affected subjects. Figure S3. Correlations between (changes in) BMI and (changes in) leptin levels (* *p* < 0.05, ** *p* < 0.01, *** *p* < 0.001). Figure S4. Correlations between baseline BMI and changes in relative and absolute ${\overset{\cdot }{V}O}_{2}\max$ (* *p* < 0.05). Figure S5. Correlation between body fat (%) at baseline and changes in relative and absolute ${\overset{\cdot }{V}O}_{2}\max$. Figure S6. Body fat (%) as assessed by skin fold calipometry throughout the intervention. Figure S7. Gender-specific differences with respect to correlations between baseline leptin concentrations and changes in relative ${\overset{\cdot }{V}O}_{2}\max$. Table S1: Body weight, body fat and waist circumference as assessed at baseline (T0), T1 and T2. Body fat was assessed using skinfold calipometry. Due to the low number of subjects for which anthropometric data were assessed at T1, *p* values were only calculated for differences between T0 and T6.
