# Supplementary material for: Adipokines as Predictive Biomarkers for Training Adaptation in Subjects with Multimorbidity—A Hypothesis-Generating Study
Source: J Clin Med. 2023 Jun 29;12(13):4376. doi: 10.3390/jcm12134376 (PMC10342946; doi:10.3390/jcm12134376)
Supplement: Supplementary file 1 [file jcm-12-04376-s001.zip › jcm-2436144-supplementary.pdf]

Suppl. Figure S1

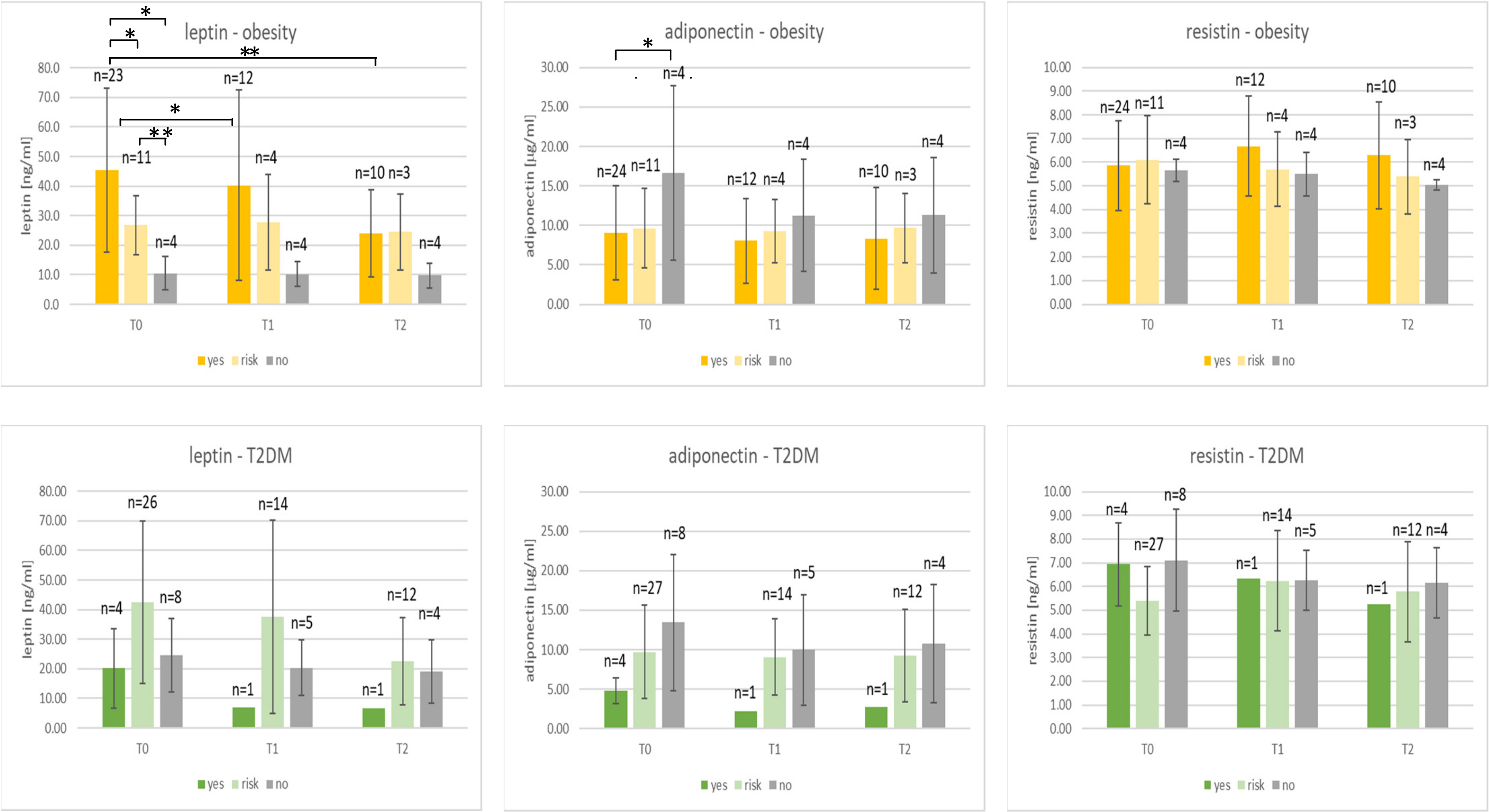

Suppl. Figure S2

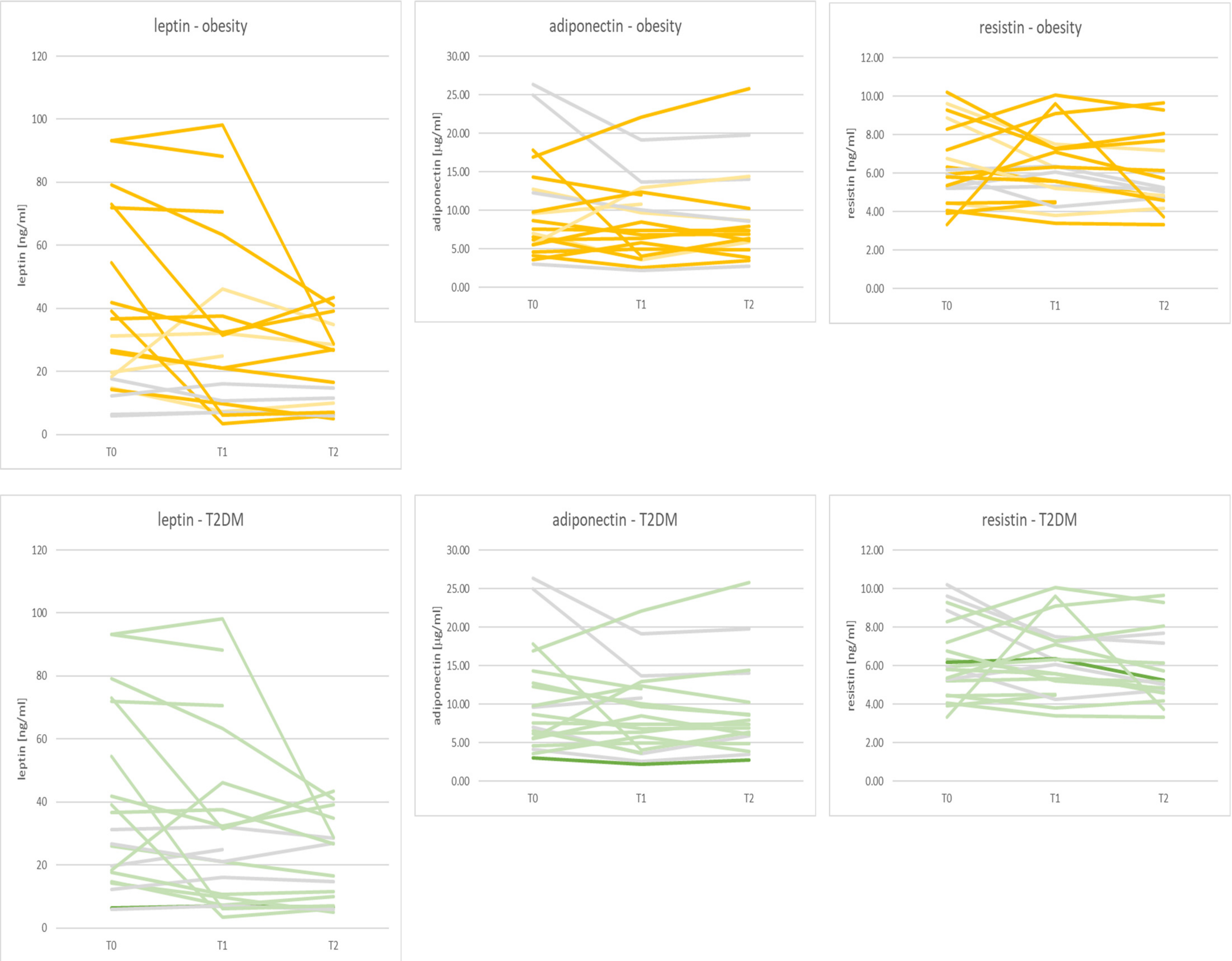

Suppl. Figure S3

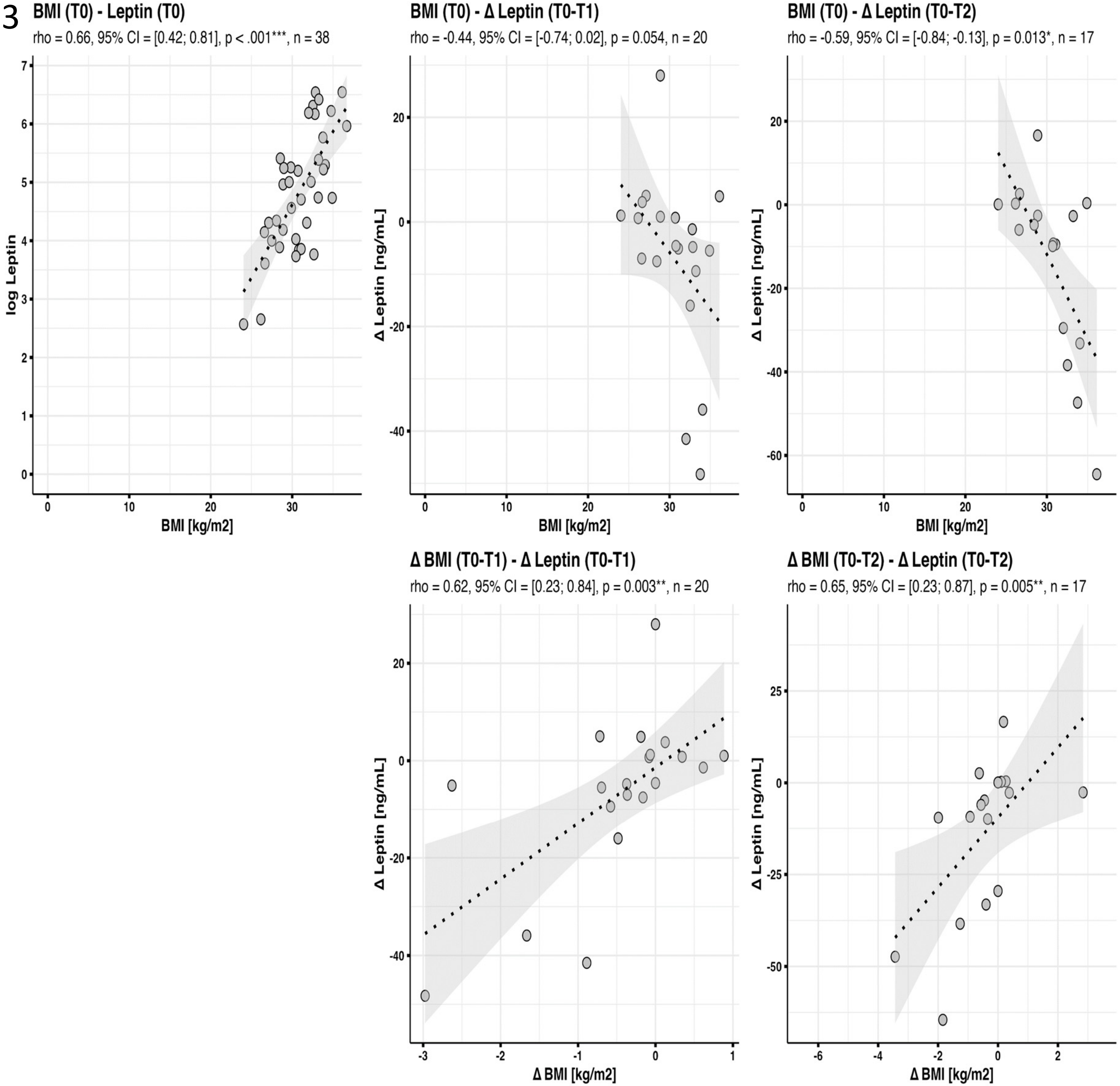

Suppl. Figure S4

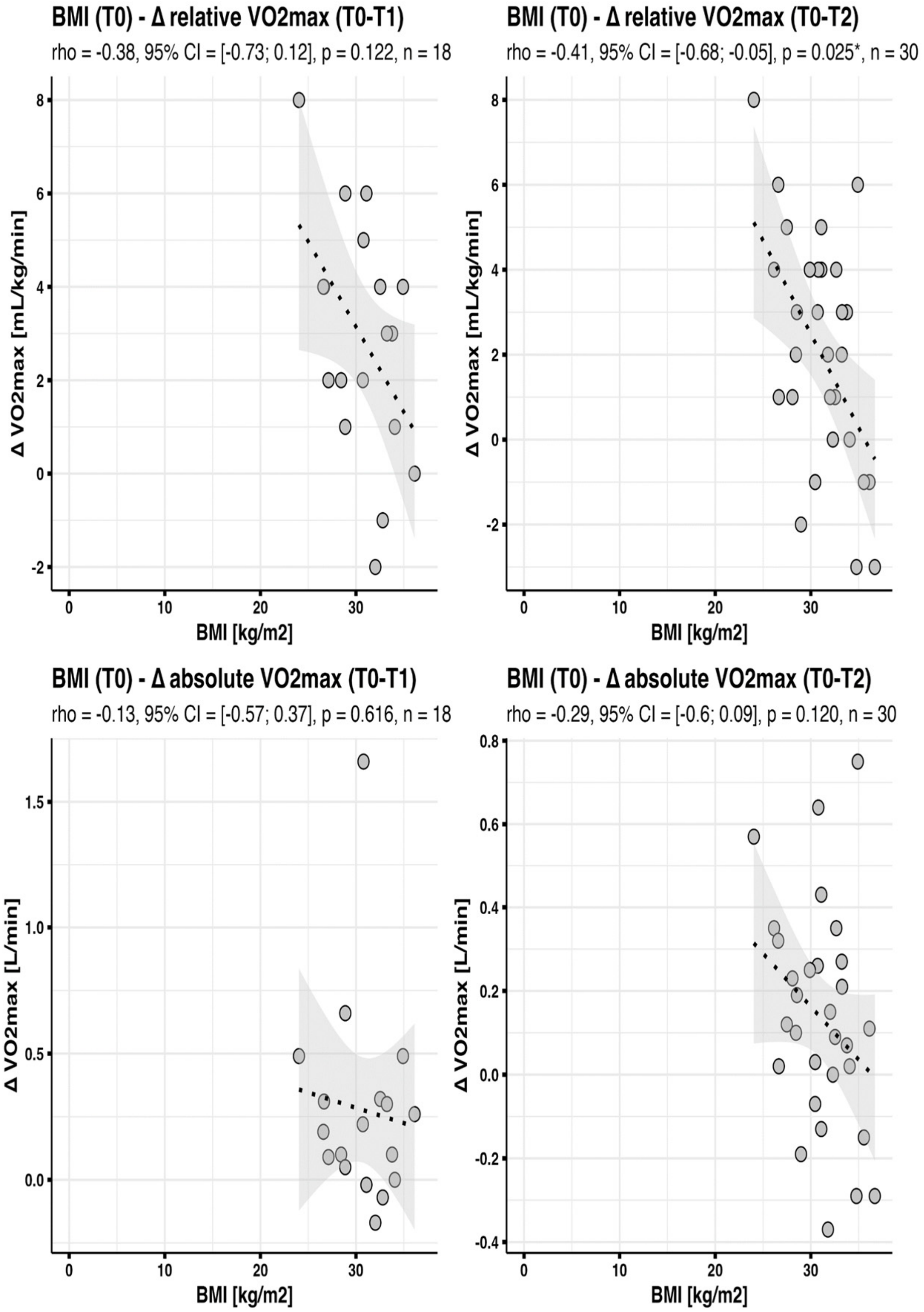

Suppl. Figure S5

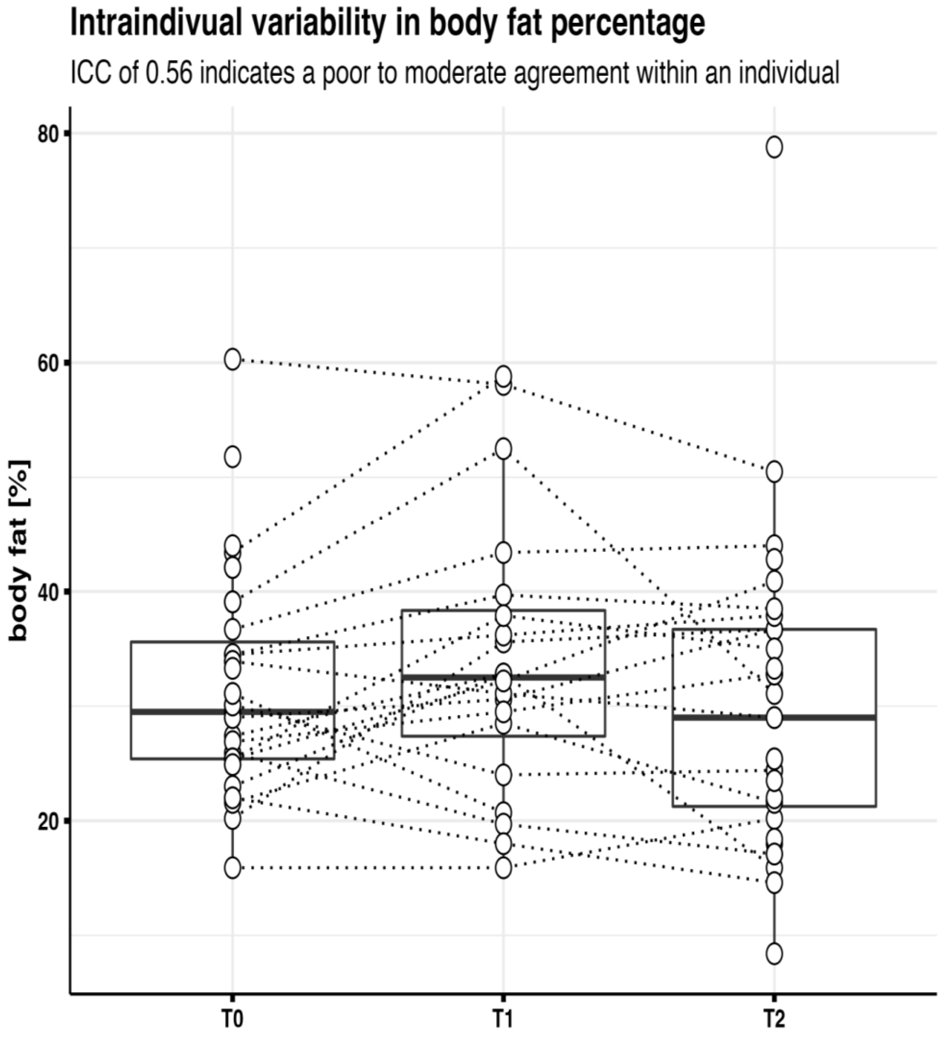

Suppl. Figure S6

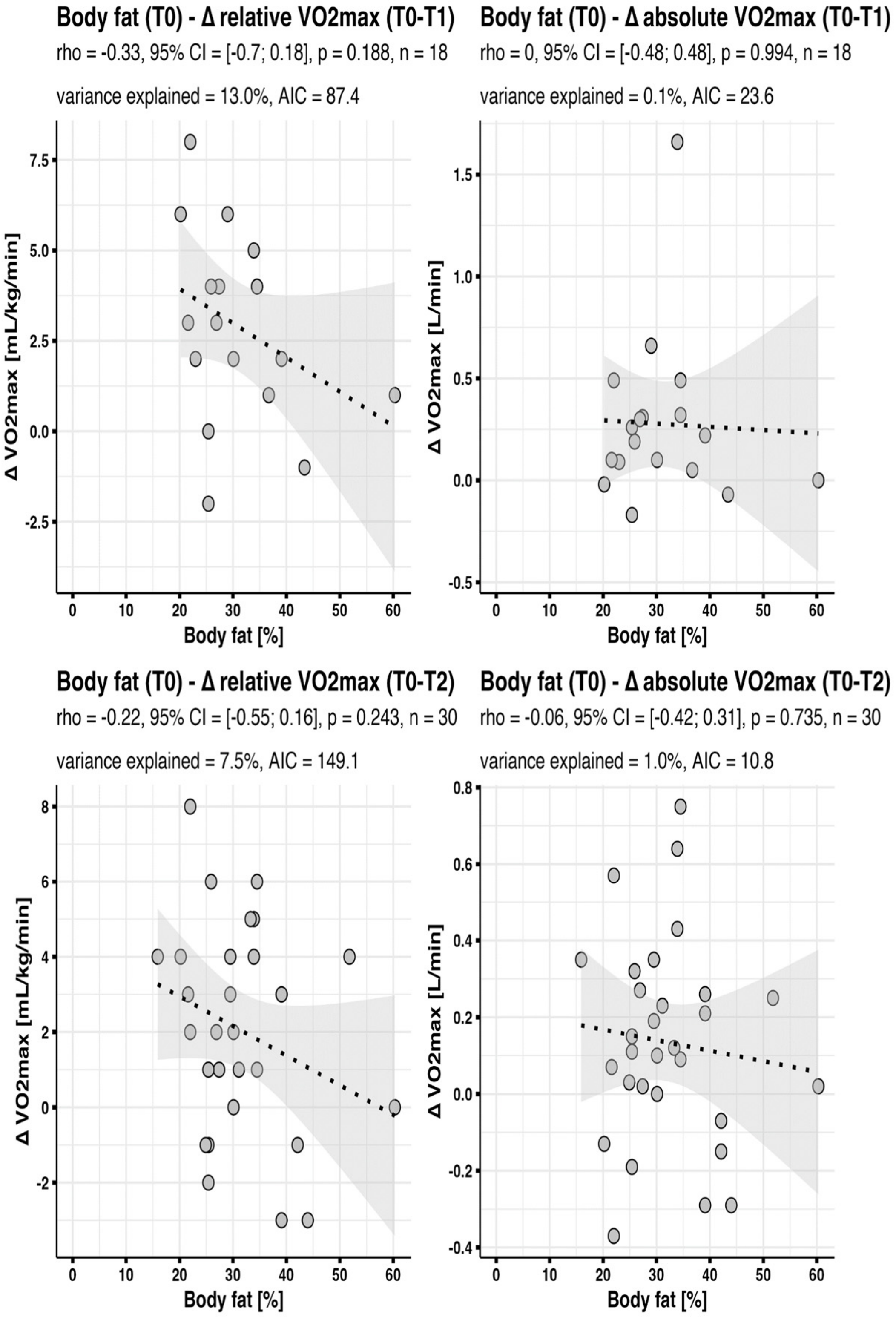

Suppl. Figure S7

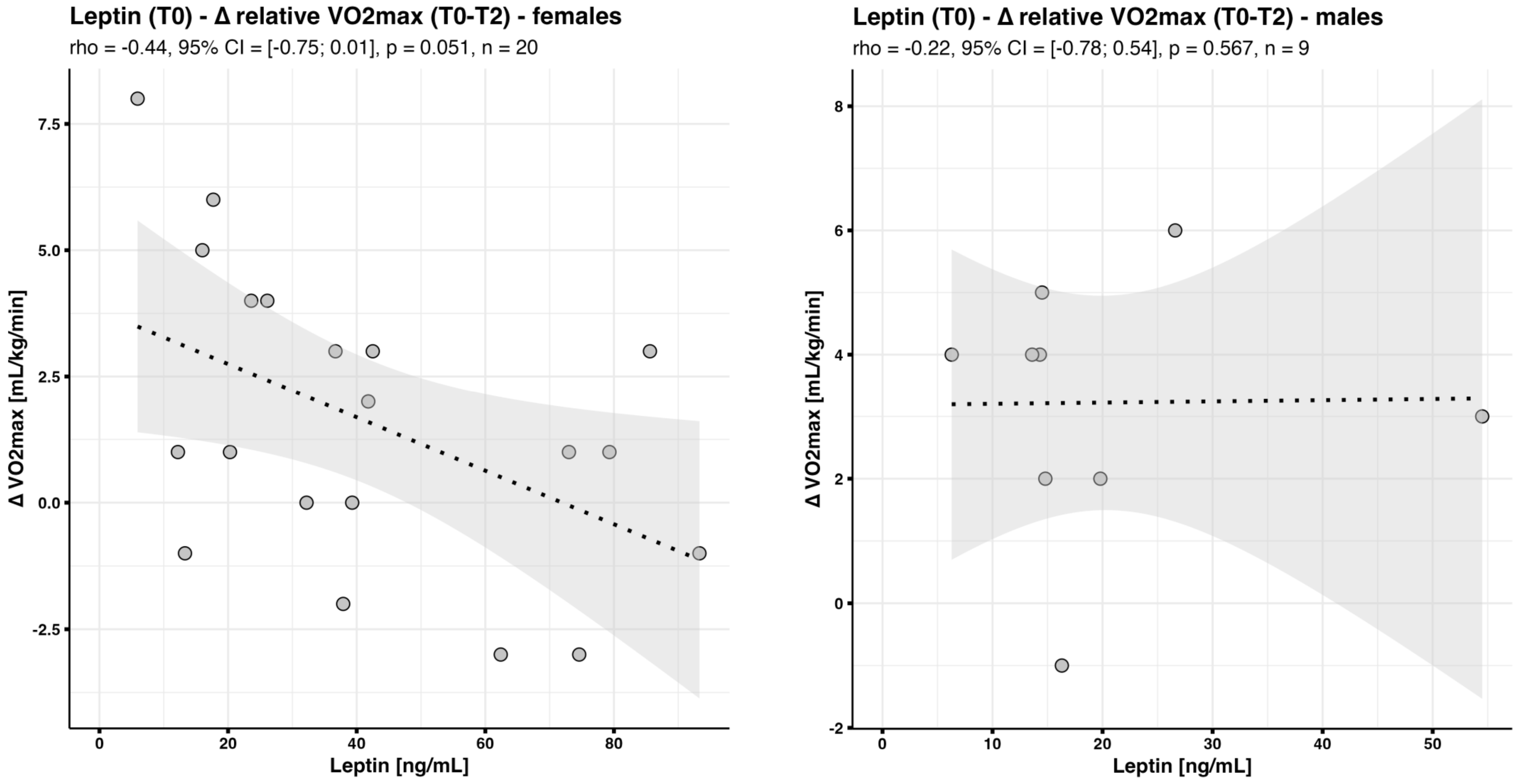

Suppl. Table S1

	Body Weight [kg]		Body Fat [%]		Waist circumference [cm]	
	Mean	SD	Mean	SD	Mean	SD
t0 (n=39)	90.8	12.4	31.6	9.0	110.1	14.1
t3 (n=20)	88.9	14.7	34.0	12.2	106.4	11.6
t6 (n=32)	89.1	13.0	30	13.3	105.3	10.7
n=32	p= 0.08		p= 0.3		p= 0.08	
